# Breastfeeding in times of COVID-19: a scoping review

**DOI:** 10.1590/1980-220X-REEUSP-2021-0556en

**Published:** 2022-06-15

**Authors:** Silvana Regina Rossi Kissula Souza, Ana Paula Pereira, Naiane Ribeiro Prandini, Ana Clara Antunes Pereira Resende, Efigênia Aparecida Maciel de Freitas, Tatiane Herreira Trigueiro, Marilene Loewen Wall

**Affiliations:** 1 Universidade Federal do Paraná, Departamento de Enfermagem, Curitiba, PR, Brazil.; 2 Universidade Federal do Paraná, Faculdade de Enfermagem, Curitiba, PR, Brazil.; 3 Universidade Federal do Paraná, Programa de Pós-Graduação em Enfermagem, Curitiba, PR, Brazil.; 4 Universidade Federal de Uberlândia, Faculdade de Medicina, Uberlândia, MG, Brazil.

**Keywords:** Postpartum Period, Breast Feeding, COVID-19, Review, Periodo Posparto, Lactancia Materna, COVID-19, Revisión, Período Pós-Parto, Aleitamento Materno, COVID-19, Revisão

## Abstract

**Objective:**

to identify how the COVID-19 pandemic has influenced postpartum women in breastfeeding.

**Method:**

a scoping review, with a search in seven databases. Studies available in full, in English, Portuguese or Spanish, published from December/2019-April/2021 were included. The analysis was carried out by categorizing common themes.

**Results:**

25 studies were included, grouped into five categories, presenting the influence of the pandemic: in the routine of breastfeeding care, evidencing preventive measures against COVID-19; in breastfeeding rates, highlighting changes in dietary practices; in the support network for breastfeeding, indicating a lack of service care; in the postpartum women’s emotions, with predominance of concern and stress; in the use of technology to support breastfeeding, with teleservice facilitating care.

**Conclusion:**

the COVID-19 pandemic has influenced new forms of care, in the offer and duration of breastfeeding, in emotional health and in the support network fragility. It is expected to contribute so that health professionals provide care with greater assertiveness in the face of this new situation.

## RESUMO

**Objetivo:**

identificar como a pandemia de COVID-19 tem influenciado as puérperas no aleitamento materno.

**Método:**

*scoping review*, com busca em sete bases de dados. Incluíram-se estudos disponíveis na íntegra, em inglês, português ou espanhol, publicados de dezembro/2019-abril/2021. A análise deu-se por categorização de temas comuns.

**Resultados:**

incluíram-se 25 estudos, agrupados em cinco categorias, apresentando a influência da pandemia: na rotina de cuidados ao aleitamento materno, evidenciando medidas preventivas contra COVID-19; nas taxas do aleitamento materno, destacando mudanças nas práticas alimentares; na rede de apoio para o aleitamento materno, apontando falta de assistência de serviços; nas emoções das puérpera, predominando preocupação e estresse; no uso da tecnologia para apoio ao aleitamento materno, com teleatendimento facilitando a assistência.

**Conclusão:**

a pandemia de COVID-19 influenciou novas formas de assistência, na oferta e no tempo de amamentação, na saúde emocional e na fragilidade da rede de apoio. Espera-se contribuir para que profissionais da saúde proporcionem assistência com maior assertividade diante dessa nova situação.

## RESUMEN

**Objetivo:**

identificar cómo la pandemia de COVID-19 ha influido en las puérperas en la lactancia.

**Método:**

scoping review, con búsqueda en siete bases de datos. Se incluyeron estudios disponibles en su totalidad, en inglés, portugués o español, publicados entre diciembre/2019 y abril/2021. El análisis se llevó a cabo mediante la categorización de temas comunes.

**Resultados:**

se incluyeron 25 estudios, agrupados en cinco categorías, que presentan la influencia de la pandemia: en la rutina de cuidados de la lactancia materna, evidenciando medidas preventivas contra la COVID-19; en las tasas de lactancia materna, destacando los cambios en las prácticas dietéticas; en la red de apoyo a la lactancia materna, indicando falta de asistencia en el servicio; en las emociones de las puérperas, con predominio de preocupación y estrés; en el uso de tecnología para apoyar la lactancia materna, con teleservicio facilitando la asistencia.

**Conclusión:**

la pandemia de COVID-19 influyó en las nuevas formas de asistencia, en la oferta y duración de la lactancia materna, en la salud emocional y en la fragilidad de la red de apoyo. Se espera contribuir para que los profesionales de la salud brinden asistencia con mayor asertividad ante esta nueva situación.

## INTRODUCTION

The Coronavirus Disease 2019 (COVID-19) pandemic, declared on March 11, 2020, by the World Health Organization (WHO), caused by the novel Severe Acute Respiratory Syndrome Coronavirus 2 (SARS-CoV-2), emerged in Wuhan, China, late 2019, spreading across all continents, infecting and victimizing millions of people^(1,2)^, with 235,175,106 confirmed cases worldwide as of October 5, 2021^(3)^.

The first case reported in Brazil was on February 26, 2020, and as of July 17, 2021, 19,342,448 confirmed cases of COVID-19 have been recorded in the country^(4)^. Among pregnant women and postpartum women, 544 deaths were reported in 2020, and as of May 26, 2021, 911 deaths have been recorded^(5)^. Postpartum women present clinical manifestations similar to those of the general population and, for the Ministry of Health (MoH), women up to the 14^th^ postpartum day are considered a risk group for COVID-19^(2)^.

Considering the recommended social isolation, behavioral changes and uncertainties occurred, such as women experiencing their labor without a companion and postpartum women, being separated from their children after birth. The possibility of the virus being transmitted through breast milk was also a cause for concern for postpartum women, sometimes being told that breastfeeding was not safe^(6)^.

A review identified SARS-CoV-2 ribonucleic acid (RNA) genetic material in breast milk^(7)^; however, the evidence is still not clear about the potential for transmission of this virus by this route. Therefore, WHO and institutions such as the MoH^(2)^, a Brazilian Federation of Gynecology and Obstetrics Associations^(8,9)^, the Brazilian Society of Pediatrics^(10)^ and the American Academy of Pediatrics recommend continuing breastfeeding, as the practice reduces the risk of infants having severe respiratory symptoms^(11,12)^. Moreover, antibodies to SARS-CoV-2 were found in breast milk, suggesting protection against infection with the virus^(13)^. Breastfeeding protects women from many diseases, presents fewer symptoms related to emotional problems, reinforces the bond between the mother-child dyad, generating a decrease in the demand for medical care by lactating women^(14)^.

Due to the pandemic, in order to reduce the displacement of patients, in order to reduce exposure to SARS-CoV-2, there was a reorganization in health care aimed at this population, with some consultations carried out by video call, when possible, or by phone^(2,6,15)^. In the UK, postpartum women who gave birth during the lockdown due to COVID-19 had more frequent contact with a healthcare professional than those who gave birth before this period. Of the 1,049 postpartum women who gave birth before the lockdown, 57% (n = 601) reported reduced support for infant feeding in this period^(15)^. Regarding breastfeeding, some postpartum women chose not to breastfeed, however, for most mothers, breastfeeding practice remained. There were numerous behavioral changes in the routine of postpartum women^(16)^.

Faced with the problem of breastfeeding in times of a pandemic and the importance for health that this practice represents, both for children and for postpartum women, this study aimed to identify how the COVID-19 pandemic has influenced postpartum women in breastfeeding.

## METHOD

### Design of Study

This is a scoping review, based on the method proposed by the Joanna Briggs Institute (JBI). A scoping review aims to map concepts that underpin an area of research, report the types of evidence available, regardless of methodological quality, and identify existing gaps in the research field^(17)^. Thus, the steps proposed by the JBI were followed, which included: the research objective and question definition; inclusion criteria development; data search approach description, selection, and extraction and presentation of evidence; search; selection; extraction; evidence analysis; results presentation; and synthesis of evidence^(17)^.

The guiding question was elaborated from the Population, Concept and Context (PCC) strategy, being defined: P - postpartum women; C - breastfeeding during the COVID-19 pandemic; C – without delimitation of scenarios. In view of this, the guiding question was formulated: how has the COVID-19 pandemic influenced postpartum women in breastfeeding?

**Chart 1.**
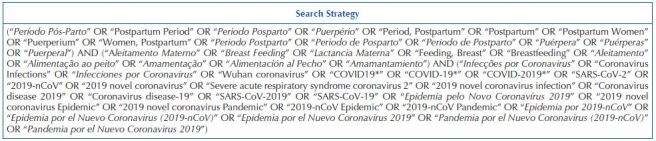
Scoping review search strategy – Curitiba, PR, Brazil, 2021.

An initial search was carried out in the Virtual Health Library (VHL) and PubMed/MEDLINE databases to analyze the terms used to describe the articles relevant to the study. Then, a search was carried out in the VHL, Cumulative Index to Nursing and Allied Health Literature (CINAHL), Embase, PubMed/MEDLINE, Scientific Electronic Library Online (SciELO), Scopus and Web of Science databases. The search strategy in Chart 1 was developed with the help of a librarian at *Universidade Federal do Paraná* (UFPR), according to the Descriptors in Health Sciences (DeCS) and Medical Subject Headings (MeSH), and the same search strategy was used in the databases that composed the study.

### Selection Criteria

Studies including postpartum women and/or puerperium, which simultaneously investigate breastfeeding and the COVID-19 pandemic, available in full in English, Portuguese or Spanish, available institutionally, primary studies, review articles, experience reports, research reports, comments and opinion articles, having been published from December 2019, the time of onset of the first cases of unidentified pneumonia that started in Wuhan, China and was confirmed to be COVID-19, to April 2021, being the period of completion of the research, were included. Editorials, letters, videos, websites, news, pre-prints, abstracts and protocols were excluded.

### Data Collection

Data collection took place from April to June 2021. Initially, the articles found were incorporated into the EndNote software, a reference manager that helps researchers in the operationalization of the selection of primary studies^(18)^. After removing the duplicates in EndNote, studies were entered into the Rayyan software, an application developed to speed up the initial screening of titles and abstracts of studies in systematic reviews^(19)^, in which the removal of duplicates that were not detected by EndNote occurred. These removed duplicates were considered only once, and not removed in their entirety.

Titles and abstracts were reviewed by a reviewer on Rayyan. In cases of doubt, the articles remained for the next phase, which involved reading in full by two reviewers independently. At this stage, the results were discussed in a consensus meeting, and conflicting studies (n = 5) were resolved by reading the entirety blindly by a third reviewer to define the insertion or exclusion of studies. To present the selection process of scoping review studies, the Preferred Reporting Items for Systematic Reviews and Meta-Analyses extension for Scoping Reviews (PRISMA ScR) was used, as recommended by the JBI^(17)^.

To extract the data from the selected studies, an instrument made available by the JBI was used^(17)^. The selected information was authorship, year of publication, title, journal, volume, edition, pages, country, language, context, participants (age and number), objective(s), methodology/methods, main results, and conclusions.

### Data Analysis and Treatment

Data analysis took place through the categorization of studies, bringing together themes in common, involving data coding, with the selection of registration units (RU), corresponding to clippings of significant and representative textual content for analysis, thus originating the themes. After that, RU are enumerated and, then, data classification and aggregation in the form of categories, consisting of the grouping of common themes^(20)^.

## RESULTS

**Figure 1.**
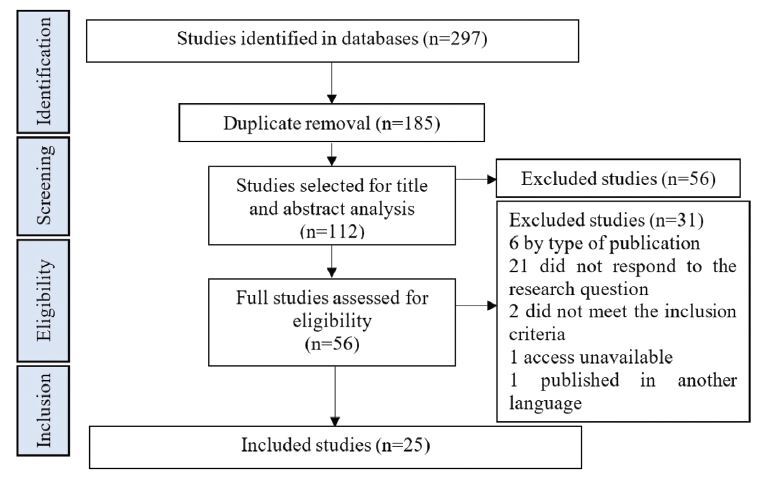
Scoping review structure flowchart. Curitiba, PR, Brazil, 2021.

The search resulted in 297 studies. After removing the duplicates, reading the title and abstract and reading in full, the final sample consisted of 25 studies, as shown in Figure 1.

Of the included studies (Table 1), there was a predominance of English (n = 22.88%), followed by Spanish (n = 2.8%) and Portuguese (n = 1.4%). The United States of America was the country with the highest number of publications (n = 9, 36%), followed by Spain, Italy (n = 3, 12%), Brazil (n = 2, 8%), China, Vietnam, Belgium, India, Nepal, Iran and Austria (n = 1.4%), in addition to a study in the SIBEN Network of Latin America and Equatorial Guinea (n = 1.4%). Articles published in 2020 predominated (n = 15, 60%), with 10 (40%) in 2021.

**Table 1.** Studies included in the scoping review – Curitiba, PR, Brazil, 2021.

Country/year	Objective	Population
USA/2021^(21)^	Build support and promotion of breastfeeding in the community, change health behavior through peer support, and address the social and economic determinants of health as a means of addressing breastfeeding as a public health imperative.	Pregnant women and postpartum women (n = 110)
Brazil/2021^(22)^	Analyze the clinical conditions for breastfeeding, which is a crucial opportunity for postpartum women and their children, in addition to preexisting guidelines on this topic. The study also observed the impacts of the SARS-CoV-2 pandemic on the affective mother-fetus dyad bond.	Postpartum women
China/2020^(23)^	Assess the clinical and immunological characteristics of mother-infant pairs affected by COVID-19, breast milk specifically tested for pathogens, SARS-CoV-2 neutralizing antibodies and immune components. Explore the feasibility of breastfeeding and related transmission possibilities.	Pregnant women and postpartum women (n = 14)
USA/2021^(24)^	Study a large sample of women who gave birth during the first spikes of COVID-19 and compare them with women who gave birth before the pandemic. Ask whether COVID-19 is associated with stressful childbirth and whether acute childbirth stress mediates the association between the presence of COVID-19 in communities and long-term post-traumatic stress and maternal bonding problems.	Postpartum women (n = 1274)
Vietnam/2021^(25)^	To describe the use of Early Essential Newborn Care (EENC) for the first childbirth of COVID-19 in Vietnam, at Hoa Vang Medical Center, a 170-bed district hospital in Da Nang City designated to manage COVID-19 cases.	Postpartum women (n = 1)
USA/2020^(26)^	Ask about current hospital practices.	Hospital institution professionals
Italy/2021^(27)^	Explore psycho-emotional distress, tested by the Edinburg Postnatal Depression Scale (EPDS) in early postpartum and breastfeeding initiation practices, defined according to the WHO among quarantined women who gave birth in a COVID-19 hotspot in northeastern Italy.	Postpartum women (n = 299)
USA/2020^(28)^	Describe Infection Prevention and Control (IPC) policies and practices for women in labor, childbirth and postpartum and for newborns (NB) of SARS-CoV-2 positive mothers who were seen at the Well Infant Nurseries (WBN) and Neonatal Intensive Care Units (NICU) and after hospital discharge, which were implemented during the first weeks of the first wave of the COVID-19 pandemic in New York, i.e. from mid-March to mid-May.	Hospital institution professionals
Spain/2021^(29)^	Determine the maternal and perinatal repercussions of pregnant women infected by SARS-CoV-2 during childbirth and clinical puerperium in several centers in the Valencian Community. The secondary objectives were to determine the absence or existence of vertical transmission and to describe the maternal care provided during the epidemic and the type of breastfeeding performed.	Pregnant women and postpartum women (n = 13)
USA/2020^(30)^	Discuss various care practices that have changed in the COVID-19 era, including the use of antenatal steroids, delayed cord clamping (DCC), mother-NB separation, and breastfeeding.	Hospital institution professionals
USA/2020^(31)^	Describe the unique experience of Infant-friendly Hospital at the epicenter of the COVID-19 pandemic.	NB of mothers tested for COVID-19 (n = 118)
Belgium/2020^(32)^	Provide estimates of SARS-CoV-2 infections among pregnant women and breastfeeding women, as well as assess women’s perceived impact of the pandemic on their breastfeeding practices, medical advice, and social support during pregnancy and lactation.	Pregnant women (n = 2,647) and breastfeeding women (n = 3,823)
USA/2021^(33)^	Explore perceptions of social support among breastfeeding mothers during the COVID-19 pandemic.	Breastfeeding women (n = 29)
Spain/2020^(34)^	Demonstrate that DCC is safe in mothers with confirmed SARS-CoV-2 infection.	Pregnant women (n = 403)
India/2020^(35)^	Present the case of a 26-year-old COVID-positive postpartum mother, separated from her premature infant at 28 weeks’ gestation, who was admitted to the NICU. In this case, the study will highlight the physical, psychosocial, spiritual concerns and stigma that she had to face during the quarantine period.	Postpartum women (n = 1)
Nepal/2020^(36)^	Assess knowledge, attitude and practice (KAP) regarding COVID-19 among postpartum women who gave birth at a tertiary care center in Bharatpur, Chitwan, and to describe their experience during hospital admission.	Postpartum women (n = 203)
Brazil/2020^(37)^	Guide the breastfeeding of mothers with suspected or confirmed COVID-19.	Postpartum women
Iran/2021^(38)^	Describe the experience of three mother-infant pairs who were offered a virtual bond during the COVID-19 pandemic in northern Iran.	Postpartum women (n = 3)
USA/2020^(39)^	Provide an overview of unintended consequences of separation policies and the harmful/harmful impacts of separation on breastfeeding.	Postpartum women
USA/2021^(40)^	Present the experience of three healthy mothers and their infants for the first time, as they gave birth in the hospital and breastfed during the beginning of the pandemic in Philadelphia.	Postpartum women (n = 3)
Austria/2020^(41)^	Assess the scientific literature available as of May 1, 2020 and discuss common questions about COVID-19 in the context of pregnancy and the postpartum period.	Postpartum women and pregnant women
Italy/2020^(42)^	Provide an overview of how care has been organized and how we are currently managing mothers and infants with suspected or proven SARS-CoV-2 infection before and after birth.	Hospital institution professionals
Spain/2021^(43)^	Review how the COVID-19 pandemic has impacted breastfeeding practices/plans and mothers’ expectations and investigate the effect that unmet breastfeeding expectations have on women’s mental health.	Postpartum women
SIBEN Network of Latin America and Equatorial Guinea/2020^(44)^	Assess and report the clinical features and outcomes of SARS-CoV-2 infection in pregnant women and their NB in Latin America.	Mothers (n = 86)
Italy/2020^(45)^	Report the type of childbirth and the immediate neonatal outcome in women infected with SARS-CoV-2 observed in the early phase of the epidemic in Lombardy.	Mothers (n = 42)

Of the 25 studies, 4 (16%) were characterized as cross-sectional studies, 2 (8%), as retrospective studies, 1 (4%), as ambispective and prospective, 6 (24%), as experience/case reports, 5 (20%), as review articles, 2 (8%), as comments, 1 (4%), as research report, short communication, case-control study and descriptive study. Of 21 studies, 16 (76%) were in hospitals, 4 (19%) in the community and 1 (5%) in primary care. Postpartum women (n = 11, 44%), pregnant women and postpartum women (n = 4, 16%), hospital institution professionals (n = 4, 16%), mothers (n = 2, 8%), pregnant women and breastfeeding women, breastfeeding women, pregnant women and NB of mothers tested for COVID-19 (n = 1, 4%) participated in the study.

From the categorical analysis, common themes emerged that answer the research question, organized into five categories: *Influence of the pandemic on breastfeeding care routine*; *Influence of the pandemic on breastfeeding rates*; *Influence of the pandemic on the support network for breastfeeding practice*; *Influence of the pandemic on postpartum women’s emotions*; *Influence of the pandemic on the use of technology to support breastfeeding*.

### Influence of the Pandemic on Breastfeeding Care Routine

Studies have pointed out preventive measures adopted against COVID-19^(22,23,25–31,35–45)^. They highlighted the recommendation to use a mask when breastfeeding^(22,23,25,28,30,36,37,41,42,45)^, hand hygiene before touching the infant^(22,25,28,30,36,37,41,42)^ and when handling the breast pump^(22,28,37,41,42)^ and the adoption of tests for COVID-19 at hospital admission^(26,28,30)^.

Regarding the change in the hospital routine, maintenance of rooming-in with a distance of at least 1 meter between the crib and the mother’s bed was mentioned^(22,25,26,28,29,31,37,41,42,44)^. Cases were highlighted in which rooming-in was not allowed or was discouraged^(26,29–31,37,41,42,44)^. Concern about exposure to the virus, together with the prevention measures adopted, such as the dyad separation, can harm breastfeeding^(30,37,39,41,43)^.

It was observed that breastfeeding was maintained and supported with a discussion between the mother and the health team about the risks and benefits^(28,35,37,41,42,45)^. However, it was described that breastfeeding was not possible, due to the dyad being separated^(30,35,42)^.

Regarding the presence of a companion, the permission of follow-up during the postpartum period^(29,37)^, the non- permission^(42,44)^ and the non-receipt of visits during hospitalization were described^(37,42)^. Regarding the visits of parents to hospitalized infants, it was recorded that the mother was authorized to visit her child after 16 days of hospitalization^(38)^ and that visits were not allowed^(42)^.

The COVID-19 pandemic influenced lactation care and hospitals started to offer virtual breastfeeding consultations^(26)^. Breastfeeding classes were canceled, as postpartum women sought friends instead of health professionals^(40)^.

Fragility of information related to COVID-19, pregnancy and puerperium was observed^(29,35–39,41,43,44)^, negatively affecting childbirth, puerperium and breastfeeding management in women positive for COVID-19^(29)^, mental health^(35)^, humanized care^(44)^ and the challenge of making a decision regarding breastfeeding due to lack of information^(38)^.

On the other hand, the knowledge about COVID-19, demonstrated by postpartum women^(36,40)^, helped them keep their children protected from the SARS-CoV-2 virus^(36)^, and the benefits of breastfeeding encouraged the maintenance of this practice^(40)^. A study showed that the non-performance of breastfeeding, due to the dyad separation, can bring negative outcomes to postpartum women, such as increased risk of postpartum hemorrhage, maternal anemia, spaced births, increased risk of breast cancer, among others, in addition to damage to infants’ health^(39)^. Finally, it was highlighted that rooming-in and breastfeeding practice allowed postpartum women to learn about safe prevention measures against COVID-19 and breastfeeding or milk extraction, reducing the risks of transmission of the virus^(31)^.

### Influence of the Pandemic on Breastfeeding Rates

In this category, the use of formula feeding was described^(23,42)^, due to NB being in the NICU during isolation^(23)^ or parents being quarantined^(42)^. It was found that mothers positive for COVID-19 had higher rates of formula feeding, 56.8% (n = 71), followed by pumped breast milk, 36% (n = 45)^(43)^ and 63% (n = 49) opted for the formula, compared to 24% (n = 19), who maintained breastfeeding, and 13% (n = 10), pumped breast milk^(44)^.

Breastfeeding was performed during postpartum hospitalization of mothers with COVID-19^(23)^ during NB’s hospitalization in the NICU^(28)^ and until postpartum women was asymptomatic, thus being able to establish breastfeeding^(38)^. Breastfeeding during postpartum hospitalization was reported^(25–27,31,34,45)^. In Italy^(27)^, the rate of exclusive breastfeeding was lower (n = 107, 70.39%) in postpartum women who gave birth during the COVID-19 pandemic, compared to postpartum women who gave birth in 2019 (n = 123, 86.39%). It was observed that, of 1,343 hospitals, the rate of exclusive breastfeeding decreased in 12.2% of them^(26)^.

In one study, 94% (n = 31) of NB who were with their mothers were breastfed in the first hour of life^(31)^. The breastfeeding rate in a group that had DCC was higher compared to the early cord clamping group (77.3% vs. 50.2%)^(31)^. A study^(45)^ pointed out that 26.2% (n = 11) of postpartum women with COVID-19 breastfed in the postpartum period.

Exclusive breastfeeding, breast milk and expressed breast milk^(40)^ were reported. A study observed that, of 97% in breastfeeding, 53% of infants were exclusively breastfed at home^(26)^. It was identified that 55% of mothers provided breast milk exclusively during meetings of the breastfeeding support group, in addition to the increase in the breastfeeding rate from 43% in 2017 to 55% in 2020^(21)^.

There was a record of lower rates of exclusive breastfeeding of mothers who breastfed during the pandemic, adopting more complementary feeding practices, as well as switching from formula milk to breastfeeding due to lack of formula, cost, fear of contamination of the formula or because they believe that milk was the best option to protect their children Mothers mentioned a positive impact of the pandemic on breastfeeding^(43)^.

Infant feeding plans were also mentioned, with a one-year feeding plan combining breast milk on the breast and pumped^(40)^. It was pointed out that the infant’s diet did not change due to the pandemic and that 97% of mothers did not consider stopping offering breast milk, as well as women with previous breastfeeding experience reported that SARS-CoV-2 had no influence on how they dealt with breastfeeding^(32)^.

The protection that breast milk can offer against SARS-CoV-2 was also mentioned, demonstrating that, of four samples, three tested positive for IgM or IgG against SARS-CoV-2^(23)^. It is noteworthy that decreased breastfeeding could limit protection against the virus^(39)^.

Studies have mentioned an increase in the frequency of breastfeeding^(32,43)^, demonstrating growth compared to before the pandemic, one of the reasons being at home longer due to the lockdown and the desire to offer protection against the virus through breast milk, considering extending the time of breastfeeding due to the coronavirus^(32)^. Increased maternity leave duration was reported as a positive influence on the journey to breastfeed, as well as the fear of lack of formula milk, being an incentive to continue breastfeeding^(33)^.

Moreover, decreased frequency^(43)^ has been described. Postpartum women mentioned that the decline or interruption of breastfeeding was due to the consequences of the lockdown, such as working from home alongside other childcare responsibilities, an increased workload or a reduction in milk production due to coronavirus concerns^(32)^.

There was a record about the refusal of postpartum women (n = 3, 23.1%) to breastfeed, even wishing to do so, after knowing the positive result for COVID-19^(29)^ and that, at some point, there was interruption of breastfeeding^(40,43)^ and change of feeding to milk in formula^(43)^.

### Influence of the Pandemic on the Support Network for Breastfeeding Practice

Themes related to this category correspond to the support received by postpartum women both from health professionals and from support groups, family, friends and co-workers^(21,26,30,32,33,35,37,40,43)^. The support network involving family and friends^(33,40)^ and co-workers^(33)^ was cited. The care of health professionals included care and guidance in breast- feeding care and consultation on lactation^(40)^. In a hospital, videoconferencing was observed with families who could not go to visits^(30)^. The positive influence of a breastfeeding support group was reported^(21)^, but there was a cancellation of support groups due to the pandemic^(40)^.

The lack of care by health services was observed^(26,32,33,43)^. There was a reduction in access to face-to-face lactation support^(26)^. Postpartum women reported dissatisfaction with the care they received, in addition to lactation support reduction in the hospital environment^(33)^. They felt the impact on medical advice, reported that they received less professional care compared to before the pandemic. Mothers who breastfed for less than 6 weeks reported that the pandemic affected their care and less than 10% of postpartum women reported having received more professional care^(32)^.

Lack of family support was reported during postpartum women’s hospitalization with COVID-19^(35)^. There was a report of the desire to have received more support from daycare centers and the family that, due to the pandemic, could not be close^(33)^. Of 39% of postpartum women who reported having received less social support during breastfeeding, 87% reported having family and friends^(32)^.

### Influence of the Pandemic on Postpartum Women’s Emotions

The studies gathered in this category presented the feelings expressed by postpartum women. Concerns due to the pandemic were described^(33,35,40,43)^, attributed to the return to work due to new policies that make it difficult to remove breast milk at work, related to the maintenance of lactation^(33)^ with regard to both manual milking of breast milk and extraction by extracting pumps, for not being able to perform maternal functions because it is far from their hospitalized children, with judgments due to the disease^(35)^, with the financial burden to buy infant formula and its availability in stores, as well as if stress would affect the supply of milk^(40)^ and with the transmission of the virus through breastfeeding^(43)^.

A study identified that 23.03% (n = 35) of postpartum women who gave birth during the COVID-19 pandemic had the Edinburgh Postnatal Depression Scale (EPDs) score for risk of depressive symptoms >12, compared to 11.56% (n = 17) of postpartum women who gave birth in 2019, as well as presenting higher scores on the anhedonia and depression subscale. It was also observed that postpartum women who were exclusively breastfed had significantly lower scores in these subscales than those who established complementary practices and artificial feeding^(27)^.

Appetite loss and interest in daily activities were reported during hospitalization^(35)^ and depressive symptoms in postpartum women breastfeeding^(43)^. From another perspective, postpartum women in exclusive breastfeeding had lower EPDS scores, compared to other feeding methods^(43)^.

Stress^(24,33,35,40,41,43)^ was related to the breastfeeding trajectory due to lack of support and how the pandemic could influence postpartum women and their son^(33)^, also due to unfulfilled maternal duties^(35)^. Frustration^(33,40,43)^ was described as a disappointment with the social distancing measures, for having planned the participation of her family and friends in her birth, and, due to the pandemic, only her husband could be present^(40)^. Stress was also related to expectations of an unmet maternity and the feeling of missed opportunity to breastfeed their children^(43)^.

Fear was reported by the possibility of infection from hospitalization or breastfeeding^(35)^ and the diagnosis and consequences that this can cause^(37)^. There were reports of anxiety due to concerns^(43)^, fear^(35)^, feelings of isolation^(33,35)^, guilt^(35,40)^, sadness for not receiving visits from family and friends during hospitalization^(37)^ and for not participating in support groups due to social distancing^(43)^.

The challenges described were related to decision-making regarding breastfeeding with limited information about COVID-19^(38)^ and the fact that if the pandemic had occurred in the first experience of postpartum women, the challenges would have been greater^(33)^. Another aspect pointed out was that the mother-child bond may end up being impacted by preventive measures adopted to combat COVID-19, in addition to the fear and anxiety by mothers that may end up affecting the construction of this bond^(22)^. It was also reported the gratifying moment of postpartum women to have contact with their child after being unable to visit them for days, thus being able to start maternal care^(38)^.

### Influence of the Pandemic on the Use of Technology to Support Breastfeeding

Here are included studies that reported the experiences of postpartum women with the use of technology^(21,31,33,36,38,40)^. The sources of information described were the online environment^(40)^, such as social networks^(33,36)^, television and radio^(36)^. Regarding online consultations, after hospital discharge, there was 100% compliance with televisits^(31)^ and postpartum care facilitated through telehealth, including lactation visits^(40)^. However, there was difficulty and concern in obtaining good care related to breastfeeding through telehealth^(33,40)^.

The use of video calls in a hospital was mentioned, being carried out by professionals between postpartum women and NB, who were hospitalized in another unit, making it possible to send photos and updates about the infant until postpartum women could visit them. Virtual visits helped the mother to express her milk to send to her child^(38)^. Also noteworthy is the increase in the frequency of meetings of a breastfeeding support group after the beginning of the pandemic, in the online format. Social media was a good alternative to involve participants, considering continuing with the virtual option of meetings, since, in this format, it is possible for mothers who have difficulty attending in person to participate^(21)^.

## DISCUSSION

Changes in breastfeeding practices demonstrated^(22,23,25,28,30,36,37,41,42,45)^ are in line with the recommendations of WHO^(46)^, Centers for Disease Control and Prevention (CDC)^(47)^ and MoH^(48)^, in order to prevent transmission of the virus to the infant. The maintenance of rooming-in^(22,25,26,28,29,31,37,41,42,44)^ and breastfeeding^(28,35,37,41,42,45)^ corroborate the WHO recommendations^(11)^. The dyad must remain together, and breastfeeding must continue, regardless of suspicion or confirmation of COVID-19.

The CDC^(49)^ advises that the decision for rooming-in be made in consensus between the mother and the team, after knowing the risks and benefits, since this practice helps in the development of mother-infant bond and in breastfeeding. As mentioned in a study^(50)^, only 12% of postpartum women who were separated from their children during hospitalization in the COVID-19 pandemic breastfed at home, compared to 27.8% of mothers who were not separated, demonstrating that separation has a negative impact on breastfeeding, such as observed in the findings^(27,30,35,37,39,41–43)^.

WHO recommends the presence of a companion during labor and childbirth^(51)^. In Brazil, mothers are entitled to a companion of choice during labor, childbirth and immediate postpartum, regulated by law^(52)^, however, in two studies in this review, postpartum women did not have this permission^(42,44)^. The presence of a companion, in addition to conveying security to postpartum women, was associated with good care practices, such as breastfeeding in the first hour of life, choosing the childbirth position, not being tied up, submission to non-pharmacological maneuvers and analgesia for pain relief^(53)^, highlighting the importance of companions in this process.

The impact of COVID-19-related misinformation^(30,36–39,41,43,44)^ on postpartum women who stopped breastfeeding is observed, in which they were more likely to be told by health professionals or family and friends that breastfeeding would not be safe or that they could not do so if they had symptoms of the disease^(6)^. It is possible to see the importance of transmitting updated information about COVID-19, as evidence about the virus that causes this disease changes rapidly. In this way, health professionals must guide and make decisions, in a shared way, with the family, respecting parents’ will^(1)^. In this sense, a scientifically based strategy to support breastfeeding women was observed by lactation consultants, in which real-time online meetings were promoted with professionals from various health areas, in order to resolve doubts from families and so that postpartum women remain encouraged to continue breastfeeding, reinforcing for postpartum women the importance of consulting information about COVID-19 and breastfeeding from safe and reliable sources^(54)^.

Regarding formula feeding, a study found data similar to the findings^(23,42–44)^, demonstrating that this type of feeding predominated in the group of mother-infant dyads who were separated during hospitalization, being 81.6% compared to 27.8% of dyads that were not separated. Breastfeeding rate was lower among separated dyads (0%) compared to non-separated dyads (22%)^(50)^. The results found on exclusive breastfeeding^(21,26,27,32,40,43)^ are corroborated by studies, in which 58.6% of participants breastfed exclusively at home during the pandemic^(6)^, in which 40% of 316 mothers who gave birth during social isolation were exclusively breastfed at home^(15)^, and who identified that, at discharge, 69.4% of infants were exclusively breastfed, compared to 97.7% of infants in 2018^(55)^.

It was observed in the findings of this review that most meal plans were not negatively influenced by the pandemic^(32,40,43)^. In the literature, 219 (14.2%) parents reported changes in diet due to the pandemic, with 95% weaning later than planned^(56)^. However, in one study, 35.3% of mothers changed their meal plans due to COVID-19, considering the separation and, consequently, the difficulty of latching on to the breast as reasons^(50)^. Mothers who gave birth during the lockdown reported that feeding plans changed due to lack of breastfeeding support (6.6%), mainly in face-to-face care, with practical problems, such as latching on to the breast, which resulted in the withdrawal of breast milk and introduction of formula or interruption of breastfeeding^(15)^.

There is a record of the presence of IgM or IgG antibodies against SARS-CoV-2 in breast milk^(23)^. In the review, there were two evidences of the presence of IgG antibodies against the same virus in breast milk, but no IgM antibodies were identified^(7)^. These findings indicate possible protection for infants against SARS-CoV-2, and reduced breastfeeding could limit protection against the virus^(39)^.

The increase in the frequency of breastfeeding^(32,33,43)^ is in accordance with data obtained in the UK and USA, in which one of the reasons for this increase among postpartum women was being at home longer during the pandemic^(15,56)^. Despite this, there have been reports of decline, interruption or discontinuation of breastfeeding^(29,32,43)^. These results are consistent with another finding, in which insufficient professional support, latching difficulty, tiredness, insufficient milk and pain were attributed to stopping breastfeeding in the UK in the current pandemic^(6)^.

Still in the same study, 67% of participants reported feeling less support for breastfeeding during the lockdown^(6)^, another finding similar to this review^(29,32,33,43)^. It was noted, in a study carried out before the current pandemic, that adequate support for breastfeeding plays a fundamental role in the perceived success of breastfeeding, in which the absence of breastfeeding difficulties and receiving support in the event of difficulties was associated with a lower risk of non-exclusive breastfeeding^(57)^. Women who sought professional help due to breastfeeding difficulties were more likely to discontinue exclusive breastfeeding^(58)^. Also, some postpartum women, during the COVID-19 pandemic, stopped breastfeeding earlier than they wanted due to lack of support in the hospital and at home^(59)^.

As for the support network^(32,33,35)^, there is a study that recorded that support for infant feeding has decreased since the lockdown in 57% of women who gave birth before the lockdown, as well as support for day care has decreased for 69% of participants^(15)^. Furthermore, the lack of social and emotional support in the pandemic has negatively impacted the breastfeeding experience among postpartum women in the UK^(6)^, reinforcing the importance of social support for breastfeeding women, observed in the positive influence of the support group on breastfeeding^(21)^. Social support has strong protection against postpartum depression (PPD), and having an adequate support network is critical to reducing PPD symptoms^(60)^.

Regarding the influence on emotions^(27,35,43)^, psychophysiological factors such as stress and sleep can affect milk production^(61)^. In a survey in Belgium, of 5,866 women, 2,421 of whom were pregnant and 3,445 breastfeeding women, almost half of the sample had depressive or anxious symptoms. The data obtained was considerably higher than the estimates obtained before the COVID-19 pandemic^(62)^. Another study carried out before the pandemic identified an association between maternal satisfaction with breastfeeding and PPD symptoms, in which there was a higher prevalence of satisfaction with breastfeeding among postpartum women without PPD symptoms^(63)^.

Social isolation, decreased social support, financial problems are related to an increased risk of developing anxiety and mood disorders^(64)^, this fact was evidenced in our findings^(33,35,37,38,40,41,43)^. Concern and guilt were identified, evidenced in a study in the USA, due to the limited supply of breast milk, as a result of increased stress for postpartum women due to the pandemic^(65)^. Changes arising from the pandemic, such as social distancing, can negatively affect postpartum women’s experiences and emotional state, increasing their risk of developing mental health problems^(12,15)^.

As seen in studies carried out during the pandemic, in Serbia, 14.8% of postpartum women demonstrated a risk for non-psychotic postpartum anxiety and mood disorders, related to social distancing, emotional problems and lack of social support^(66)^. Research carried out in five European countries with 9,041 women, of which 5,134 are breastfeeding women, identified depressive symptoms in 13% of breastfeeding women and rates of moderate to severe generalized anxiety symptoms in 10% of breastfeeding women^(67)^.

In addition to the negative effects of not breastfeeding on women’s health described in one of the studies^(39)^, breastfeeding mothers have lower levels of anxiety, negative mood, stress, as well as prolonged sleep patterns and better chances of a secure mother-child bond^(68)^. The literature also shows that the link between the dyad and, consequently, breastfeeding, can be impacted by social distancing measures due to the COVID-19 pandemic, such as the mother-child separation^(69)^.

The use of technology, such as online care, has become essential nowadays in order to limit exposure to SARS-CoV-2, thus being able to guarantee continuous access to health care^(64)^. Additionally, remote care enables the promotion of breastfeeding and facilitates access to health services in distant locations^(70)^. This is also in line with tech-related observations^(21,26,31,38,40)^. However, telelactation requires planning and participation from both professionals and parents so that the service is adequate^(71)^.

Before the COVID-19 pandemic, telelactation was practiced, with good acceptance of videoconferencing for lactation consultations among postpartum women^(72)^. Breastfeeding consultants, in which 73% of the care was provided online in the current pandemic, reported that breastfeeding women were notably satisfied with the service provided, mainly because they had a reduced support network, enabling breastfeeding promotion and breastfeeding women’s mental health^(54)^.

The limitations for this scoping review consist of not having published a prospective protocol, not performing manual searches in the main journals or other information sources, and the restriction to Spanish, English and Portuguese.

## CONCLUSION

The articles analyzed in this study pointed out that the influence of the COVID-19 pandemic on breastfeeding practice can be very heterogeneous, since for some postpartum women, it can be said that the pandemic had a positive influence, allowing more time for breastfeeding, while for others, the opposite was demonstrated, interrupting breastfeeding due to greater burden or less social support. It was understood that safe information, social support, emotional health and new ways of caring for lactation are factors to be considered in breastfeeding care. Thus, the support network of postpartum women during the COVID-19 pandemic has become an important issue for future research.

This review also pointed out that telehealth can be a good alternative for support in this period, allowing the transmission of safe guidelines, and can also identify other problems not related to breastfeeding. It is hoped that this study will bring new knowledge to postpartum women and society in general and to health professionals, in order to contribute to care with greater assertiveness in the face of this new situation, promoting care that meets the new needs in relation to breastfeeding.

## ASSOCIATE EDITOR

Ivone Evangelista Cabral
